# A Manual of the Principles and Practice of Ophthalmic Medicine and Surgery

**Published:** 1847-01

**Authors:** 


ART. IX.—*A Manual of the Principles and Practices of Ophthalmic Medicine and Surgery.* By T. WHARTON JONES, F.R.S., Lecturer on Anatomy, Physiology, and Pathology, at the Charing-cross Hospital, &c. With four copper-plates, coloured, and ninety woodcuts.—*London,* 1847. 8vo, pp. 570.

THE object of the author in writing this book, and its general character, are so concisely set forth in the preface that we cannot do better than transcribe a portion of it.

"To produce a work on diseases of the eye which should serve at once as a text-book for students, and a book of reference for practitioners, has been the great aim of the author in composing this Manual. Accordingly, besides carefully discussing the principles, he has laboured to give such practical exposition of the subjects as will be found available at the bedside of the patient, and in the operating-room. At the same time, he has not neglected the opportunity which the subject offers, of illustrating the general doctrines of pathology, especially those of inflammation. . . . . . . The author thinks it proper to mention, that he has incorporated in the present volume the various contributions to ophthalmic medicine and surgery, some anonymously, in the course of the last fifteen years, and also that he has freely availed himself of the information contained in the principal works, British and Foreign, on the subject."

We are confident that the reader will find, on perusal, that the execution of the work amply fulfils the promise of the preface, and sustains in every point the already high reputation of the author as an ophthalmic surgeon as well as a physiologist and pathologist. The book is evidently the result of much labour and research, and has been written with the greatest care and attention; it possesses that best quality which a general work, like a system, or manual can show—viz., the quality of having all the materials whencesoever derived, so thoroughly wrought up and digested in the author's mind as to come forth with the freshness and impressiveness of an original production. We regret that we have received the book at so late a period as precludes our giving more than a mere notice of it, as although essentially and necessary a compilation, it contains many things which we should be glad to reproduce in our pages, whether in the shape of new pathological views, of old errors corrected, or of sound principles of practice in doubtful cases clearly laid down. But we dare say most of our readers will shortly have an opportunity of seeing these in their original locality, as we entertain little doubt that this book will become what its author hoped it might become, a manual for daily reference and consultation by the student and the general practitioner. The work is marked by that correctness, clearness, and precision of style which distinguish all the productions of the learned author.

